# Dental Attendance in Undocumented Immigrants before and after the Implementation of a Personal Assistance Program: A Cross-Sectional Observational Study

**DOI:** 10.3390/dj6040073

**Published:** 2018-12-14

**Authors:** Martijn Lambert

**Affiliations:** Department of Community Dentistry and Oral Epidemiology, Special Needs in Oral Health, Ghent University, 9000 Ghent, Belgium; Martijn.Lambert@UGent.be; Tel.: +32-494-89-64-42

**Keywords:** undocumented immigrants, oral health care, community health workers

## Abstract

Undocumented immigrants are a high-risk social group with low access to care. The present study aims to increase awareness and dental attendance in this subgroup, assisted by community health workers (CHW). Starting from 2015, two trained dentists volunteered to perform free oral health examinations and further dental care referral in a welfare organisation in Ghent, Belgium. In 2016 and 2017, a two-day oral health training was added, enabling social workers to operate as community oral health workers and to provide personal oral health advice and assistance. Over the three years, an oral health examination was performed on 204 clients from 1 to 69 years old, with a mean age of 36.7 (SD = 15.9), showing high levels of untreated caries (71.6%; *n* = 146) and a Dutch Periodontal Screening Index (DPSI) score of 3 or 4 in 62.2% of the sample (*n* = 97). Regarding dental attendance, the total number of missed appointments decreased significantly, with 40.9% in 2015, 11.9% in 2016 and 8.0% in 2017 (*p* < 0.001). Undocumented immigrants can be integrated into professional oral health care. Personal assistance by community health workers might be an effective method, although this requires further investigation.

## 1. Introduction

Undocumented immigrants are a very vulnerable social subgroup, consisting of a considerable number of people trying to remain undiscovered by local authorities. In contrast to asylum seekers and recognised refugees, they do not have a residence permit to stay legally in the country. Their estimated number varies between 7% and 13% of the total number of immigrants with an official residence permit [1]. In Belgium, there were 1,214,605 legal immigrants on the 1 January 2014, which means that the number of undocumented immigrants probably lies between 85,000 and 160,000, corresponding to approximately 1% of the total Belgian population [2].

Although epidemiological data on the oral health of undocumented immigrants are scarce, some authors previously described the oral health and oral health needs of refugees in general [3,4,5]. According to these publications, oral diseases are highly prevalent in refugees and care provision is impeded by several barriers. It can be assumed that the oral health conditions of undocumented immigrants and their access to care are comparable or even worse, because they cannot register for an official health care insurance. However, according to the United Nations International Bill of Human Rights (1966), every individual has the right to “urgent medical care”, including dental care, whether he or she has a residence status or not. Belgium ratified this universal human right and integrated it into its legislation in 1996.

In Belgium, the medical assistance provided to undocumented immigrants is financed by the federal government and organised at city level, and care can be both preventive and curative. To apply for it, people have to meet three criteria: they have been living for longer than three months in the country without permission to stay, they live in the city in which they apply for help, and they do not have a substantial income. In addition, a registered doctor or dentist needs to confirm the need for medical treatment.

Since the organisation of urgent medical care occurs at city level, there are local differences in care provision between different cities. In Ghent, Belgium, undocumented immigrants can obtain a “medical card”, allowing them to receive medical treatments for a three month period, which can be repeated for as long as the three previously mentioned conditions are met. The medical card covers every treatment which is reimbursed by Belgian Social Security. Regarding dentistry, the medical card has two main shortcomings: it does not cover tooth extractions for people under the age of 53, nor provision of a removable denture for people under the age of 50.

The present survey originates from a purely voluntary-based project in “De Tinten”, an organisation providing material and social assistance to illegal immigrants in Ghent, Belgium. In 2015, the organisation started to refer its clients to local dental clinics, driven by the high demand for dental care. However, the initial rate of missed appointments was so high (9 out of 22) that further collaboration between the organisation and the local dentists was put at stake.

In order to improve the system of referral and to increase dental attendance, the organisation set up a collaboration with researchers from Ghent university in 2016. To reduce barriers between both care providers and care demanders, the involvement of community health workers (CHW) was proposed. The involvement of CHWs in primary care showed to be an efficient way to guide underprivileged individuals towards preventive care and social services, reducing resource utilisation and community costs [6]. CHWs can also play an active role in oral health care. Benzian et al. composed a global competency matrix for oral health, involving many health professionals and groups in society, including CHWs [7]. Since oral health care provision in Belgium is almost exclusively founded upon the shoulders of the dentist, a CHW can be a valuable intermediary in oral health promotion and referral to oral health care. Greenberg et al. demonstrated the positive impact of dental case managers on Medicaid beneficiaries’ (low-income individuals) use of dental services and the number of dentists participating in the Medicaid program [8].

The present survey aims to describe the preliminary results of referring undocumented immigrants to the dental practitioner, assisted by community health workers (CHW). Apart from reporting the oral health status of the participants, the main hypothesis is the following: Is the proportion of undocumented immigrants missing their appointment with external dentists the same before and after providing personal assistance?

## 2. Materials and Methods

The present cross-sectional study, which was based on annual convenience samples, describes the evolution in the proportion of missed dental appointments in undocumented immigrants in Ghent, Belgium, from February 2015 to December 2017. The study was carried out in “De Tinten”, an organisation providing material and social assistance to undocumented immigrants in the city of Ghent.

Starting from February 2015, two trained dentists volunteered to perform free oral health examinations and dental interviews on a two-weekly base in a separated room in the organisation building. After oral consent, clients were interviewed on age, nationality and smoking habits. Nationalities were grouped according to the world health organization WHO (world health organization) regions. Individual oral health parameters included D_3_MFT, which is the total number of decayed (at cavitation level), missing (due to caries) and filled teeth [9]. Based on the D_3_MFT scores, a restorative index (RI = (FT/(D_3_ + FT)) × 100) and treatment index (TI = (M + FT)/(D_3_ + M + FT) × 100) were calculated in order to gain insights regarding the level of care. Severity of untreated dental caries was assessed using PUFA index, counting the number of teeth with visible pulp exposure, ulcerations, fistula and abscesses [10]. The plaque index of Sillness and Löe was used to measure the amount of dental plaque [11]. Periodontal health was assessed by using the Dutch Periodontal Screening Index (DPSI) for participants older than 15 years old. This index describes the severity of periodontal disease (attachment loss around the teeth) and the need for further treatment on a scale from 0 to 4, after sounding the gums with a periodontal probe [12]. After the oral health examination, clients received a professional referral letter, as well as a dental goody bag, containing a toothbrush and toothpaste. Clients could apply for new toothpaste and a toothbrush every 8 weeks, even without oral health examination or referral. Before getting an appointment with an external dental clinic, all clients were required to obtain a “medical card” from the Ghent Social Welfare Organisation, confirming their undocumented status and allowing them to receive further medical care. Accordingly, only participants without a residence permit were included in the survey.

Starting from August 2016, the two-weekly oral health examinations remained unaltered, but the medical setting of the organisation changed, by training volunteers from the organisation to operate as community oral health workers. The training was held during a two-day program, and included provision of essential information on the normal development and anatomy of human dentition, oral diseases, preventive oral health, dental administration, motivational interviewing and case management. It was performed by two university researchers (one dentist and one psychologist), providing theoretical knowledge, clinical images, practical exercises and cases using an interactive PowerPoint presentation. In addition to the educational program, participants could consult and rehearse all information on a website (www.iedersmondgezond.be), which was specifically designed for the CHWs. As part of the website, a registration system was designed to follow up dental appointments. The information of this registration system was protected by a central log-in and password, and allowed the CHWs and the organisation to receive and send text messages when a client had a dental appointment in the upcoming 24 h.

The main task of the CHWs was to increase dental awareness and dental compliance among the undocumented immigrants, by completing all necessary administrative steps prior to the first dental visit, and by following up the further appointments. After the initial oral health examination, one of the CHWs called a local dentist to make an appointment in consultation with the client. When the appointment was made, it was registered in the digital registration system. Subsequently, the head of the organisation’s medical service and the CHW considered whether the client needed personal assistance on the day of the appointment or not. The decision was made based on the linguistic capacities, personal competences and special needs of the individual client. In cases where doubt existed, or for first time visitors, personal assistance was always provided. When this personal assistance was required, the CHW received an expense allowance of €20 to cover transport and other direct costs.

At the end of the oral health examination, a referral letter was given to the client in case of need for further care. Clients were referred to the closest available dental office from their home address. When personal assistance was organised, a copy of the referral letter and the medical card of the client were given to the CHW in a closed envelope, in case the client lost or forgot it on the day of the appointment. If a second appointment was needed or the external dentist wanted to communicate directly with the organisation, the information was inserted in the closed envelope and returned to the organisation.

All external dentists were visited by the head of the medical service or contacted by phone before the first referral, in order to provide them with more information about the organisation and the involvement of CHWs. The dentists were informed and assured that the organisation would cover tooth extractions for people under the age of 53, which are not reimbursed by the government. Additionally, the dentists were allowed to apply for a “no show fee” in case of a missed appointment, which was also provided by the organisation.

The total number of appointments with external dentists was counted for 2015, 2016 and 2017. For each of these dental visits, the organisation registered whether the client was present or not. When an appointment was missed, the client was called by phone to ask for the reason for non-attendance. If the appointment was cancelled within the last 24 h, it was also considered as a missed appointment. Both the CHWs and the external dentists were asked to always contact the organisation in case of a missed appointment. When contacting the organisation, the external dentist could apply for a “no show fee” which was paid by the organisation. This fee ranged between €30 and €50, according to the dentist’s standards.

Data analysis was carried in IBM SPSS Statistics V25.0 (SPSS Inc., Chicago, IL, USA). After explorative data analyses, differences in proportions were examined using crosstabs and Chi-square statistical tests. Alpha was set at 0.05.

The study was approved by the Ethics Committee of the University Hospital Ghent (B670201526486). All subjects gave their informed consent for inclusion before they participated in the study. They received a referral letter and were supported to visit a dentist when further care was needed. Clinical data were stored in a database specifically designed for the survey, using VTiger CRM system 7.1.0RC. The data, including personal data, were protected by an external hosting company and could not be consulted or modified by a third party. Before data analysis, all records were encrypted to ensure anonymity.

## 3. Results

Over the three years, an oral health examination was performed on 204 clients from 1 to 69 years old, with a mean age of 36.3 (Standard Deviation (SD) = 15.9). Baseline characteristics are indicated in Table 1. Untreated tooth caries were visible in 71.6% (*N* = 146) of the participants (D_3_ > 0). From those with tooth decay, 46.7% had at least one tooth with visible pulpal exposure. The level of care was low, with an average restorative index of 30.3% (SD = 36.9) and a treatment index of 51.5% (SD = 37.9). Periodontal health was poor, with 62.2% (*N* = 97) of the clients having a DPSI score of 3 or 4.

**Figure 1:**
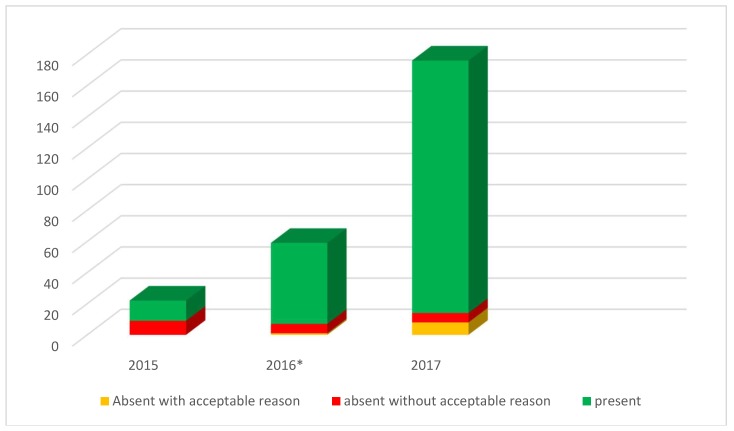
The number of appointments made with external dental practitioners and the proportion of missed appointments (y-axis) over the three study years (x-axis). *In August 2016, the personal assistance program was implemented.

Regarding dental attendance during the survey period, Figure 1 and Table 2 illustrate the number of external appointments provided to the target population for 2015, 2016 and 2017. The avoidable missed appointments without acceptable reason are indicated in red (Figure 1), the others in green. The orange bar indicates the number of missed appointments with legitimate reasons.

According to Table 2, the organisation registered 176 appointments with 16 different external dental practices in 2017, of which 89 were first dental visits. Physical assistance was provided for 87 appointments. Over the three years, the total number of missed appointments decreased significantly, with 40.9% in 2015, 11.9% in 2016 and 8.0% in 2017 (*p* < 0.001). The percentage of avoidable missed appointments dropped to 3.4%.

## 4. Discussion

The cross-sectional survey presented annual convenience samples within a Belgian organisation for undocumented immigrants. The participants were mainly young, with a mean age of 36.3. Their initial oral health care needs are considerable, as seen in Table 1. The presence of tooth decay was very high, with untreated decay at cavitation level (D_3_ > 0) being present in 71.6% of the records, of which half had visible pulp exposure. Periodontal health was very poor, especially taking into account the mean age of 36.7, with 62.2% (*N* = 97) of participants having a DPSI score of 3 or 4. The bad periodontal condition can be partially explained by the high number of smokers (46.2%). Although the present oral health outcomes are alarming, they should be interpreted with caution. The present survey used a convenience sample, which means that the observed findings cannot be generalized for all undocumented immigrants, not even within the organisation. It can be assumed that people with high dental needs will be more likely to accept the offer of a dental screening and further referral, leading to selection bias and partially declaring the high level of tooth decay and periodontal disease in the initial examinations. No information was available from non-responders.

In contrast to the high dental need, the initial care level was almost negligible before the intervention. Although the present survey cannot draw conclusions on the causes of care avoidance, several determinants might play a role. First of all, the illegal character of the participants’ residence forces them to hide from most official institutions. To get medical care, they need to present themselves to the local authorities. Although no one can ever be arrested while seeking help in one of these centres, officially registering for (oral) health care can still be a barrier. Furthermore, living in precarious conditions might also have an influence. As one of the most underprivileged groups in society, people without a residence permit suffer from the same social determinants which are mainly associated with deprived oral health: material deprivation, educational attainment, origin, professional status, and the lack of a social network. These factors are largely described in international literature as predictors of adverse oral health outcomes [13,14]. Since the average time of living in Belgium was almost five years in the presented sample, it is very likely that the adverse living conditions have played an important role in the oral health outcomes.

To support the target population, the intervention aimed to enable social workers, having a close relationship with the undocumented immigrants, to be involved in oral health care as community health workers. Since community health workers are people known by the target population, providing food and other assistance, they might have more authority and get more trust than an external caregiver. Acting as a contact person between care demander and care provider, the community oral health workers also consider the barriers perceived by the local dentists. Emphasis was put on adequate information and translation, and on the reduction of administrative burden and the number of missed appointments. Bedos et al. previously reported that frustrations expressed by dentists mainly concern missed appointments, difficulties in performing non-covered treatments and low government fees [15]. In our intervention, every dentist was called or visited personally by the head of the medical service of the organisation. During this conversation, the dentist could express his personal expectations and concerns, which were taken into account by the organisation and the CHWs. For example, when a dentist indicated not to speak French or English, the organisation only sent clients who were able to speak Dutch, or who were accompanied by a Dutch-speaking translator. The personal approach and mutual empathy resulted in a considerable network of 16 dentists accepting the undocumented immigrants in their dental practice. This can be considered a success, since the number of general dentists in Flanders is decreasing and dental practitioners are ageing. In 2017, the estimated number of qualified dentists was 1 per 1147 residents in Belgium and 1 per 1182 in Flanders (http://www.dekamer.be/QRVA/pdf/54/54K0138.pdf, p261). Furthermore, Belgium has until present no experience with dental hygienists as part of the professional dental team. When the number of dentists decreases, it can be assumed that the “law of demand and supply” will not be beneficial to vulnerable subgroups in society, such as undocumented immigrants.

Aiming to cope with the high unmet oral health needs and various user-side and supplier-side barriers, one of the major strengths of the present intervention was the creation of a safe and reliable partnership between the organisation and both the target population and the local dentists. Concerning the undocumented immigrants, the key elements of the intervention were outreach work, personal assistance and the involvement of existing networks. Indeed, oral health behaviour and oral health in general are largely affected by social networks and social support, defined as people’s “social capital” [16].

Although the first results of the involvement of CHWs suggest a reduction in barriers towards dental care provision in both undocumented immigrants and local dentists, the effects might not be automatically applicable. Care provision strongly depends on national policy and health care budgets. According to a report of Cuadra, access to health care for undocumented immigrants varies between European countries [17]. Some countries, such as Belgium, only provide the minimum as set out by the UN Human Rights and specified by article 13.2 of the “Council of Europe Resolution 1509 (2006) on Human Rights of Irregular Migrants”, whereas other countries provide more or sometimes less than this minimum access to care.

Even in Belgium, the results of the intervention might differ between cities. Since medical care for undocumented immigrants is organised at city level, inequalities in care provision between cities are probable, although this requires further investigation. The medical card in Ghent is an accessible instrument to help undocumented immigrants, but it does not exist in other cities such as Antwerp or Brussels, where decisions on reimbursement depend on each individual case. It is recommendable to obtain clear and equal legislation on a national scale, to avoid inequity and possible delocalisation of undocumented immigrants from one city to another. Medical care should not be determined by place of residence.

Although the medical card in Ghent might be an easy instrument to get access to dental care, the lack of coverage for tooth extractions for people under the age of 53 is a limitation, leading to high out-of-pocket costs. For 2017, the local organisation “De Tinten” spent €2311 of its own budget on no-show fees and uncovered basic treatment costs (excluding prosthodontic treatments), and paid €1616 for personal assistance by CHWs, yielding an average cost of €22.31 per appointment. Without external charity funding, this intervention could be compromised.

In order to reduce curative treatment costs in the future, the intervention did not focus exclusively on guiding clients to the dental office, but CHWs were trained during the educational program to pay attention to preventive oral health care and oral health behaviour. Furthermore, free dental goody bags were provided on a regular basis (every eight weeks). Budgets for preventive materials amounted to €2130 in 2017 and were also paid for by charity funding. It needs further research to investigate if this intervention can lead to a reduction of the overall costs in the long term due to improved oral health outcomes.

The present survey has some important limitations to report. Regarding the positive dental attendance rates in 2017, these results should be interpreted with caution. Increased dental attendance might not automatically imply increased dental awareness, better oral health behaviours or improved oral health outcomes, especially in the long term. Furthermore, the use of the words “urgent medical care” in legislation might also impede the mindset shift from curative care and falling from one oral health problem into the other, towards more cost saving preventive care. Although the law states that “urgent medical care” can be both curative and preventive, policy makers and stakeholders tend to consider preventive check-ups, supragingival scaling and small fillings as “non-urgent”. Even some dental practitioners feel reluctant to sign the required form to confirm that their treatment was urgent. The author suggests that “necessary medical care” would be a better wording than “urgent medical care”, avoiding semantic discussions and professional neglect of painless lesions.

Secondly, the survey could not link the external appointments to the original and individual characteristics of the clients, due to technical limitations. This makes it impossible to know if there were specific personal or oral health determinants, leading to more missed appointments. Furthermore, the present survey cannot provide information on responders and non-responders within the target population. The invitation to participate in the survey aimed to be as accessible as possible to undocumented immigrants. In order not to frighten them, it was impossible to make official lists and numbers of all undocumented immigrants who could possibly be involved. Furthermore, the recruitment process was carried out over several weeks during food distribution, making it impossible to count the total number of unique clients in this setting. Nevertheless, it is possible that the invitation attracted the most motivated clients, leading to higher rates of dental attendance.

As a final limitation, the present study is not a randomised controlled trial, exploring differences in outcomes between an intervention and control group over the same study period. Comparing different convenience samples before and after a program without the use of a proper control group inevitably leads to reporting bias. The absence of a control group was due to ethical reasons. The initial rates of missed appointments in 2015 (40.9%) negatively affected the reputation of undocumented immigrants among local dentists. Since there is no dental public health system in Belgium, all residents, legal and illegal, entirely depend on care from private dentists. If a control group was used, in which the number of missed appointments would remain high during some more years, it would be possible that local dentists would turn against all undocumented immigrants because of those in the control group. In real life circumstances, dealing with extremely vulnerable human beings, this was a risk nobody wanted to take.

Despite the clear scientific shortcomings of the survey, it is difficult to assume that the dramatic decrease in missed appointments over the two years would be due to factors other than the reported intervention. Even if this was due to other factors, the present survey shows that undocumented immigrants can be integrated into regular dental care and that high levels of dental attendance can be achieved in this population.

## 5. Conclusions

The present sample of undocumented immigrants shows very poor oral health, both in terms of tooth decay and periodontal disease. However, the decreasing proportions of missed appointments indicate that undocumented immigrants can be integrated into professional oral health care. Personal assistance by community health workers might be an effective method, although this requires further investigation.

## Figures and Tables

**Table 1:** Characteristics of the examined sample.

**Total Sample**	***N* = 204**		
	**Mean**	**SD**	**Missing**
Age	36.3	15.9	*N* = 35
Years in Belgium	4.6	4.7	*N* = 0
Plaque Index	1.4	0.8	*N* = 18
DPSI *	2.6	1.1	*N* = 16
D_3_MFT	9.4	8.4	*N* = 0
PUFA	1.6	3.0	*N* = 0
Restorative Index	30.3	36.9	*N* = 39
Treatment Index	51.5	37.9	*N* = 28
Number of teeth with active caries per person	3.4	3.9	*N* = 0
Number of teeth with visual pulp exposure per person	1.3	2.5	*N* = 0
	**Valid %**	***N***	**Missing**
**Origin (WHO region)**	-	-	*N* = 11
African Region	11.9	23	-
Region of the Americas	1.0	2	-
South-East Asia Region	0.5	1	-
European Region	67.4	130	-
Eastern Mediterranean Region	37	19.2	-
Western Pacific Region	0.0	0	-
Smoker **	-	-	*N* = 20
Yes	46.2	60	-
No	52.3	68	-
Former smoker	1.5	2	-
**Gender**	-	-	*N* = 10
Male	44.3	108	-
Female	55.7	86	-
**Active tooth decay**	-	-	*N* = 0
Present	71.6	146	-
Not Present	28.4	58	-
**Visible pulp Exposure**	-	-	*N* = 0
Present	35.8	73	-
Not Present	64.2	131	-

* From those > 15 years old (*N* = 143); ** From those > 12 years old (*N* = 150).

**Table 2:** Total number of appointments for 2015, 2016 and 2017, and the proportion and explanation of missed appointments.

Appointments	2015	2016	2017
Total number of appointments	22 (100%)	59 (100%)	176 (100%)
Client present	13 (59.1%) *	52 (88.1%) *	162 (92.0%) *
Client absent	9 (40.9%)	7 (11.9%)	14 (8.0%)
Client absent without acceptable reason	9 (40.9%)	6 (10.2%)	6 (3.4%)
Client absent with acceptable reason	0 (0.0%)	1 (1.7%)	8 (4.5%)
Cancellation >24 h before appointment	-	-	4 (2.3%)
Unforeseen circumstances (arrestation, hospitalisation) Error made by organisation or dentist	-	1 (1.7%)	2 (1.1%) 2 (1.1%)

* *p* < 0.001 according to chi-square test.
